# Differential impact of spotted fever group *rickettsia* and anaplasmosis on tick microbial ecology: evidence from multi-species comparative microbiome analysis

**DOI:** 10.3389/fmicb.2025.1589263

**Published:** 2025-05-13

**Authors:** Jin-qi Wang, Tian Yu, Hong-yu Qiu, Sheng-wei Ji, Zhi-qiang Xu, Qi-chao Cui, Hai-feng Li, Wan-feng Liang, Shuai Feng, Chen-tao Fu, Xu Gao, Zhen-zhen Han, Wan-nian Tian, Ji-xu Li, Shu-jiang Xue

**Affiliations:** ^1^Agricultural College of Yanbian University, Yanji, China; ^2^College of Animal Science and Veterinary Medicine, Heilongjiang Bayi Agricultural University, Daqing, China; ^3^Animal Health and Epidemic Prevention Center, Huludao, China; ^4^College of Animal Science, Jilin Agricultural Science and Technology College, Jilin, China; ^5^Yanbian Center for Disease Control and Prevention, Yanji, China

**Keywords:** tick, *Rickettsia*, anaplasmosis, microbiota, 16S rRNA gene, microbiome analysis

## Abstract

Tick-borne diseases (TBDs) pose a significant public health challenge, as their incidence is increasing due to the effects of climate change and ecological shifts. The interplay between tick-borne pathogens and the host microbiome is an emerging area of research that may elucidate the mechanisms underlying disease susceptibility and severity. To investigate the diversity of microbial communities in ticks infected with vertebrate pathogens, we analyzed the microbiomes of 142 tick specimens. The presence of *Rickettsia* and *Anaplasma* pathogens in individual samples was detected through PCR. Our study aimed to elucidate the composition and variation of microbial communities associated with three tick species, which are known vectors for various pathogens affecting both wildlife and humans. We employed high-throughput sequencing techniques to characterize the microbial diversity and conducted statistical analyses to assess the correlation between the presence of specific pathogens and the overall microbial community structure. Pathogen screening revealed an overall positivity rate of 51.9% for *Anaplasma* and 44.6% for spotted fever group *rickettsia* (SFGR). Among the three tick species (*Dermacentor silvarum*, *Haemaphysalis concinna*, and *Haemaphysalis japonica*) analyzed, *D. sil*var*um* (the predominant species) exhibited the highest pathogen prevalence. The results indicate significant variation in microbial diversity between tick samples, with the presence of *Anaplasma* and SFGR associated with distinct changes in the microbial community composition. These findings underscore the complex interactions between ticks and their microbial inhabitants, enriching our understanding of tick-borne diseases.

## 1 Introduction

Ticks are hematophagous arthropods that primarily infest mammals, reptiles, and birds in the wild, causing serious disease in humans who are exposed to tick bites (Adrion et al., 2015; Duron and Gottlieb, 2020; Tokarz and Lipkin, 2021). Ticks play an essential role as vectors in the dynamics of vector-borne diseases (Simo et al., 2017). Hard ticks exhibit stage-specific feeding behavior, requiring a single prolonged blood meal per developmental instar (Wikel, 2013). During their multi-day attachment period, hard ticks engage in telmophagy, a cyclical process characterized by alternating anticoagulant saliva secretion and blood ingestion through the hypostome. During this time, pathogens are transmitted through saliva, which helps initiate infection in the host (Maldonado-Ruiz et al., 2019). Concurrently, symbiotic bacteria enter the host through the tick bite. In mice, it has been demonstrated that ticks replace the majority of the pre-existing commensal bacteria on the host’s skin during blood intake (Boulanger et al., 2023), and this alteration may play an important role in transmitting pathogens. The first attempts to characterize the full tick microbiome (Andreotti et al., 2011) indicated that it is complex and of varied origin. Since then, high-throughput sequencing technologies have been utilized to further study the tick microbiome (Budachetri et al., 2014; Grandi et al., 2023; Zhang et al., 2023). Currently, *16S rRNA* genes are frequently utilized as target genes for amplicon sequencing, with the V1, V3, and V4 regions being of particular value (Sperling et al., 2017). The majority of tick-borne pathogens are of significant natural epidemiological importance, including their role in Lyme disease, anaplasmosis, rickettsiosis, and tick-borne encephalitis (Dantas-Torres et al., 2012; Madison-Antenucci et al., 2020). The microbiome plays a pivotal role in regulating various physiological processes in ticks, including immunity, nutrition, and digestion (Duron et al., 2018; Zhong et al., 2024). The environment has a significant impact on tick microbiome abundance. The prevalence of tick-borne pathogens is closely related to geographic location, climate, and other abiotic factors (Van Treuren et al., 2015).

The sampling area for this study is located on the border of China, Russia, and North Korea. This region is known for its rich biodiversity and favorable climate, which fosters the proliferation of tick communities. A substantial number of migratory birds visit the area each year, making it a potential source of pathogen transmission. This phenomenon renders the region a significant public health concern. Consequently, scientists around the world are engaged in efforts to control the spread of disease by studying the microbiomes of disease vectors (Moreira et al., 2009; Wu et al., 2019; Huang et al., 2020). Of particular importance is the elucidation of the relationship between vertebrate pathogenic infections and the arthropod microbiomes of vectors. Through the analysis of a vertical transmission model in *Rickettsia raoultii*, it has been demonstrated that interactions between rickettsiae and tick microbiome components contribute to the horizontal transmission of pathogenic rickettsiae (Du et al., 2023). In *Ixodes scapularis*, infection by anamorphs disrupts midgut microbial homeostasis, thereby facilitating the colonization by these anamorphs (Abraham et al., 2017). Conversely, the microbiome of the arthropod vector significantly influences the susceptibility of pathogens to vector-borne transmission. A substantial body of evidence has demonstrated that the gut microbial homeostasis of arthropods has a significant impact on influencing the colonization of the gut by pathogens (Gonzalez-Ceron et al., 2003; Finney et al., 2015; Gall et al., 2016; Pavanelo et al., 2020). Multiple arthropod endosymbionts show varying degrees of correlation with pathogens (Novakova et al., 2017; Budachetri et al., 2018), suggesting that the vector microbiome may have a direct influence on vectorial competence. Recently, a study conducted in the border region of Yunnan, China, discovered a mosquito gut commensal that is effective in blocking mosquito-borne viruses, representing a novel method for controlling mosquito-borne viral transmission (Zhang et al., 2024). The aforementioned evidence suggests the presence of intricate interactions between the host microbiome and pathogens. Therefore, understanding the composition of the microbiome is imperative for its rational application.

The objective of this study is to analyze the bacterial community composition of tick vectors in the region at the border of China, Russia, and North Korea in order to elucidate the distribution of tick-borne pathogens in the area and to collect fundamental data for the prevention and control of tick-borne diseases.

## 2 Materials and methods

### 2.1 Tick collection, nucleic acid preparation, and high-throughput sequencing

**Figure 1:**
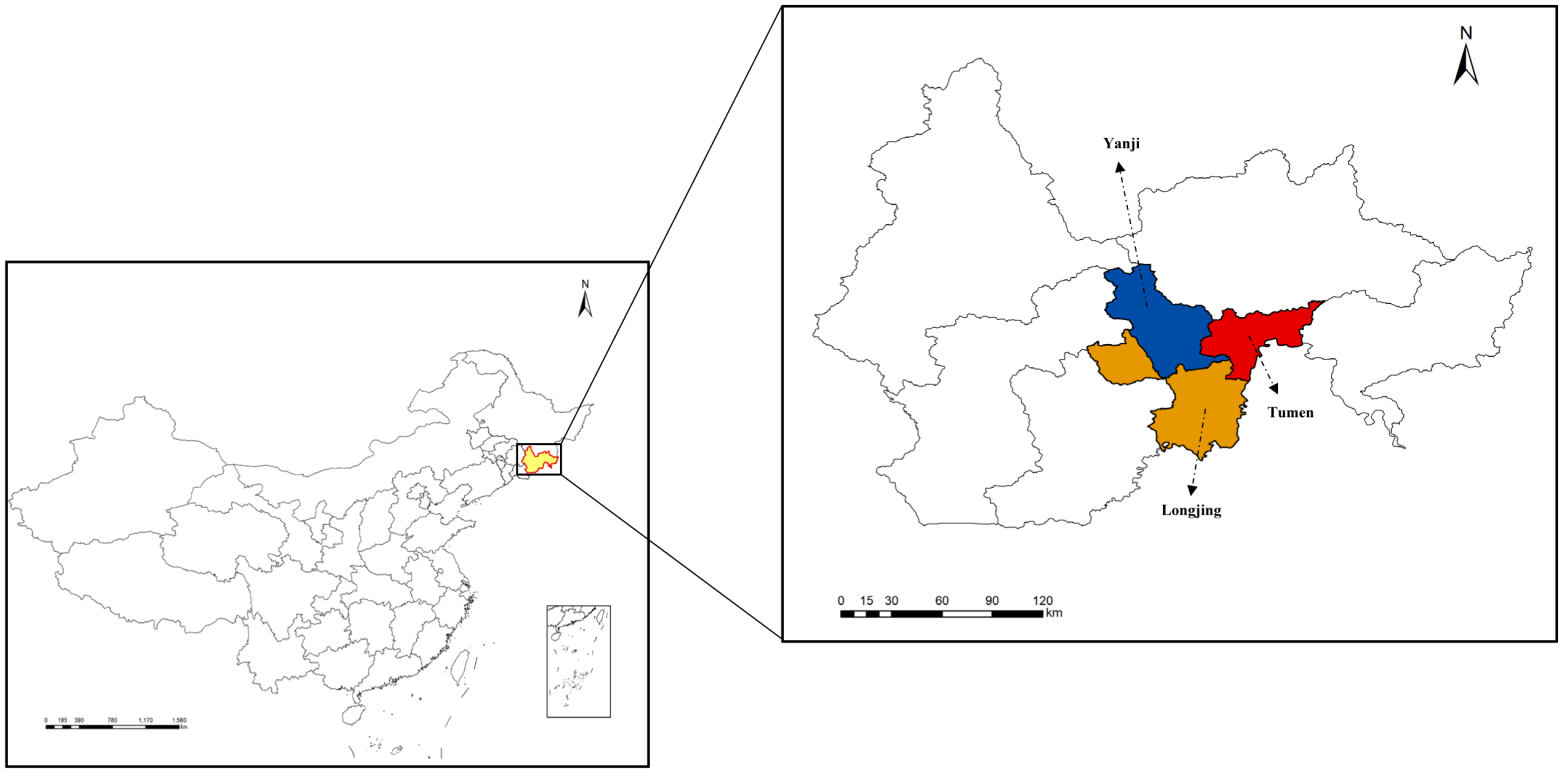
Map of the study area. Yanbian Korean Autonomous Prefecture of Jilin Province, China. Black arrows indicate the sampling locations.

Ticks were collected from April to May 2022 using standard dragging methods with corduroy cloths in low-lying scrub vegetation and forest-grassland ecotones in the Yanbian Korean Autonomous Prefecture, Jilin Province, China. Collections were conducted once a month at three sampling sites, namely Yanji (43°48′N, 129°23′E), Tumen (42°58′N, 129°50′E), and Longjing (42°46′N, 129°26′E), resulting in a total of two sampling rounds at each site (Figure 1). A total of 442 questing ticks were collected, including 326 *Dermacentor silvarum*, 54 *Haemaphysalis japonica*, and 62 *Haemaphysalis concinna* (Table 1). The collected ticks were placed in 1.5 mL centrifuge tubes based on their morphological characteristics for analysis of mitochondrial 16S ribosomal DNA (16S rDNA) gene sequences and species identification (Black and Piesman, 1994; Jia et al., 2020). The ticks were then cleaned by vortexing for 30 s in a 1% bleach solution (Binetruy et al., 2019b). This was followed by three consecutive 1-min washes using sterilized, DNA-free water. After washing, the samples were dried using sterile filter paper and stored at −80°C. Sequencing was performed on 142 single-tick pools stratified by species and sex (Table 2). Personalbio Co., Ltd. managed the entire workflow, including cryogenic homogenization in lysis buffer, DNA extraction from supernatants using DNeasy Blood & Tissue Kit (Qiagen, Hilden, North Rhine-Westphalia, Germany) amplification of bacterial 16S rRNA V3–V4 regions with primers 338F (5′-ACTCCTACGGGAGGCAGCA-3′)/806R (5′-GGACTACHVGGGTWTCTAAT-3′) (Claesson et al., 2009), library preparation with TruSeq Nano Kit (Illumina, San Diego, California, CA, United States), and paired-end sequencing (2 × 250 bp) on an Illumina NovaSeq 6000 platform (Klindworth et al., 2013; Bonnet et al., 2017). Post-sequencing, DNA aliquots were transported under cold-chain conditions for subsequent pathogen PCR validation. Sequencing datasets were classified into non-infected and infected groups based on PCR validation and species metadata, with detailed stratification criteria provided in Supplementary Table S1. The remaining tick samples were individually placed in disposable zirconia bead grinding tubes and ground using a low-temperature tissue grinding homogenizer. The genomic DNA from ticks was then extracted using a genomic DNA extraction kit (Tiangen, China).

**Table 1:** Number of tick samples and prevalence of tick-borne pathogens in this study.

Species	No. of males	No. of females	No. of infected (%)			
			SFG *Rickettsia*		*Anaplasma*	
			Male	Female	Male	Female
*D. silvarum*	149	177	48.3	55.9	61.7	66.1
*H. japonica*	17	37	17.6	18.9	11.7	16.2
*H. concinna*	39	23	33.3	17.3	23.0	17.3
Total	442		44.6		51.9	

**Table 2:** Information on tick samples for high-throughput sequencing.

Samples ID	Number	Species	Sex
DSMt 1–32	32	*D. silvarum*	Male
DSFt 33–65	33	*D. silvarum*	Female
HJMt 66–76	11	*H. japonica*	Male
HJFt 77–99	23	*H. japonica*	Female
HCMt 100–124	25	*H. concinna*	Male
HCFt 125–142	18	*H. concinna*	Female

### 2.2 Bioinformatics and statistical analyses

Microbiome bioinformatics analysis was performed using QIIME2 (v2019.4) (Caporaso et al., 2010; Rai et al., 2021). Raw sequences were demultiplexed, trimmed of primers using the cutadapt plugin, and processed through the DADA2 plugin (Callahan et al., 2016) for quality filtering, denoising, merging, and chimera removal to generate a non-singleton amplicon sequence variant (ASV) table. Taxonomy was assigned to amplicon sequence variants (ASVs) using the classify-sklearn naïve Bayes taxonomy classifier in the feature-classifier plugin (Bokulich et al., 2018) against the Greengenes 13.8 Database (DeSantis et al., 2006), excluding mitochondrial, chloroplast, and unassigned sequences, along with rare ASVs (relative abundance <0.005%) (Bokulich et al., 2013). Alpha diversity metrics (ACE/Observed/Fisher’s alpha/Shannon/Simpson/Chao1) were calculated on rarefied ASV tables (10,000 reads/sample) and compared across groups using the Wilcoxon rank-sum test with Benjamini–Hochberg false discovery rate correction. Beta diversity analysis based on Bray–Curtis distances revealed significant differences. For group comparisons, we performed Kruskal–Wallis tests with Benjamini–Hochberg correction (Benjamini and Hochberg, 2018) and PERMANOVA (Anderson, 2001). LEfSe analysis (Segata et al., 2011) identified differentially abundant taxa using Kruskal–Wallis screening (*p* < 0.05), LDA effect size >2.0, and all-against-all validation. Group-specific ASVs, identified via ASV-level Venn diagrams, were quantitatively compared between infected and non-infected groups using the Mann–Whitney *U*-test. Dominant phyla and genera (>1% mean abundance) were visualized in compositional bar plots (ggplot2), while the microbial community structure was illustrated through PCoA ordinations (QIIME2 View) (Asnicar et al., 2015).

### 2.3 Pathogen detection, phylogenetics, and prevalence analysis

The prevalence of two pathogens was detected by PCR amplification of the gene fragments of spotted fever group *rickettsia* (SFGR) (*ompA*) and *Anaplasma* spp. (16S rRNA). The sequences of the primers are shown in Supplementary Table S2. Target DNA fragments purified with a Gel Extraction Kit (Omega, Norcross, Georgia, GA, United States) were ligated into the pMD19-T vector (Takara, Japan) via TA cloning and transformed into *E. coli* DH5α competent cells (Tiangen, Beijing, China). Positive clones selected by antibiotic resistance and colony PCR underwent plasmid extraction using the Omega Plasmid Mini Kit (Omega, Norcross, Georgia, GA, United States), followed by Sanger sequencing at Jilin kumei Biotechnology Co., Ltd. The corrected sequences were then searched for similarity in the GenBank database using the National Center for Biotechnology Information (NCBI) Basic Local Alignment Search Tool (BLAST) search engine. Then, the representative sequences of the pathogens were aligned using MEGA11 software, and a phylogenetic tree was constructed using the neighbor-joining method, with the number of bootstrap replicates set to 1,000 and the Kimura’s two-parameter model. Differences in pathogen positivity rates between tick species were analyzed using Pearson’s chi-squared test, with statistical significance defined as a two-tailed *p*-value of <0.05.

## 3 Results

### 3.1 Infection of ticks with pathogens

**Figure 2:**
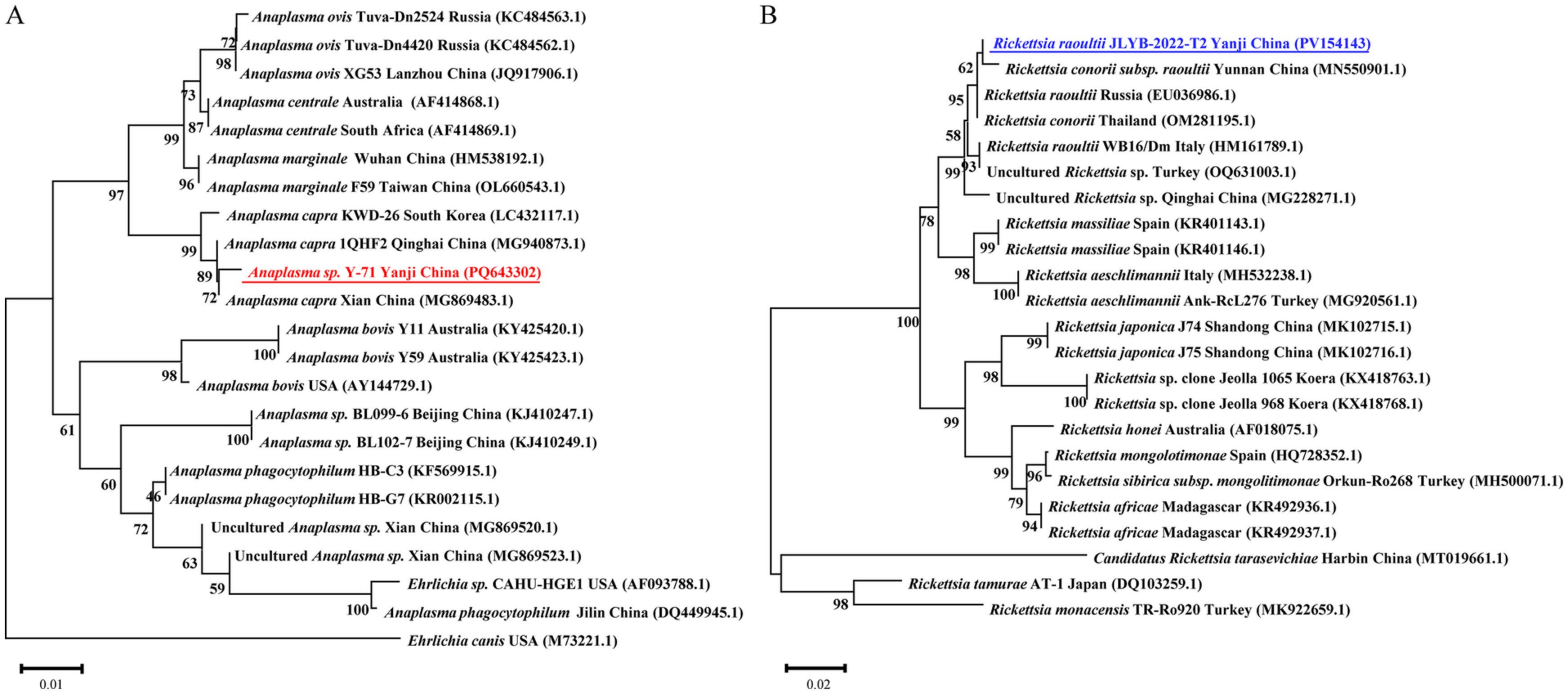
Neighbor-joining phylogenies of **(A)** *Anaplasma* spp. 16S rRNA gene sequences and **(B)** spotted fever group *rickettsia ompA*. Bootstrap support values from 1,000 replicates are indicated at branch nodes. Sequences from this study are bolded in blue (**A**; PV154143) and red (**B**; PQ643302).

PCR-based surveillance revealed an overall *Anaplasma* positivity rate of 51.9% (Table 1), with *D. silvarum* exhibiting a significantly higher infection prevalence than other tick species (*χ*^2^ = 12.302, df = 1, *p* < 0.001). For *D. silvarum*, female ticks demonstrated a marginally elevated positivity rate (66.1%) compared to male ticks (61.7%). Phylogenetic analysis of representative *Anaplasma* 16S rRNA sequences indicated 99.8–100% similarity to GenBank references, clustering in a monophyletic clade with *Anaplasma capra* isolates from South Korea (LC432117.1), Qinghai (MG940873.1), and Xian (MG869483.1), China (Figure 2A). Concurrently, SFGR showed an overall positivity rate of 44.6%, with the highest prevalence observed in *D. silvarum* female ticks (55.9%) and the lowest in *H. concinna* female ticks (17.3%). Comparative analysis of SFGR *ompA* gene sequences revealed 97.3–99.37% similarity to GenBank entries, with the closest phylogenetic proximity to *Rickettsia raoultii* from Yunnan Province, China (MN550901.1). These sequences formed a distinct clade that included *R. raoultii* strains from Russia (EU036986.1) and Thailand (OM281195.1) (Figure 2B).

### 3.2 Diversity of microbiota in ticks

**Figure 3:**
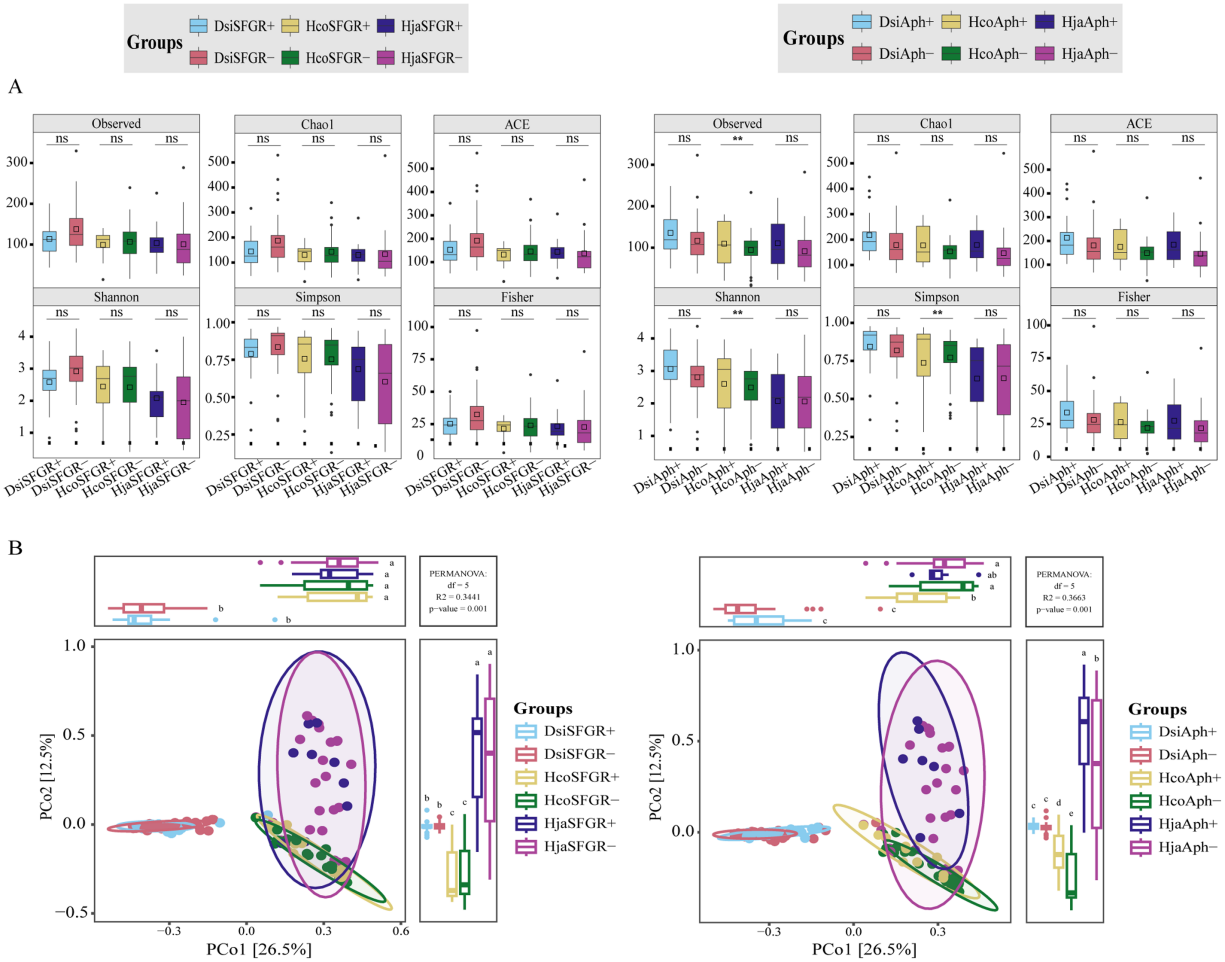
Tick microbiota diversity in pathogen-infected and uninfected groups. **(A)** α-diversity comparisons (six indices): SFGR-infected vs. SFGR-uninfected, *Anaplasma*-infected vs. *Anaplasma*-uninfected. Significance: Mann–Whitney *U*-test with Benjamini–Hochberg (^**^p < 0.01, ns: not significant). **(B)** β-diversity: Bray–Curtis PERMANOVA for each pathogen. Dsi, *Dermacentor silvarum*; Hco, *Haemaphysalis concinna*; Hja, *Haemaphysalis japonica*.

To investigate the diversity of microbial communities in ticks infected with vertebrate pathogens, we analyzed the microbiomes of 142 tick specimens. The presence of *Rickettsia* and *Anaplasma* pathogens in individual samples was determined using PCR methods. Figure 3A illustrates the distribution of alpha diversity across experimental groups. There were no statistically significant differences in alpha diversity between SFGR-infected and non-infected groups across tick species (Mann–Whitney *U*-test, *p* > 0.05). However, *Anaplasma*-infected groups exhibited significantly higher alpha diversity indices compared to their non-infected counterparts (*p* < 0.01, Benjamini–Hochberg corrected). Notably, this trend remained consistent across all three tick species investigated. Beta diversity analysis revealed a significant divergence in the microbial community structure among the tick species (PERMANOVA, *R*^2^ = 0.32, *p* < 0.001). However, congeneric *Haemaphysalis* species (*H. japonica* vs. *H. concinna*) exhibited closer clustering in the PCoA ordination based on Bray–Curtis dissimilarity (Figure 3B). Within each tick species, microbial communities showed minimal separation between experimental groups (infected vs. non-infected), indicating that pathogen exposure did not significantly alter β-diversity patterns (PERMANOVA, *R*^2^ = 0.02–0.05, *p* > 0.1).

### 3.3 Composition of microbiota in ticks

**Figure 4:**
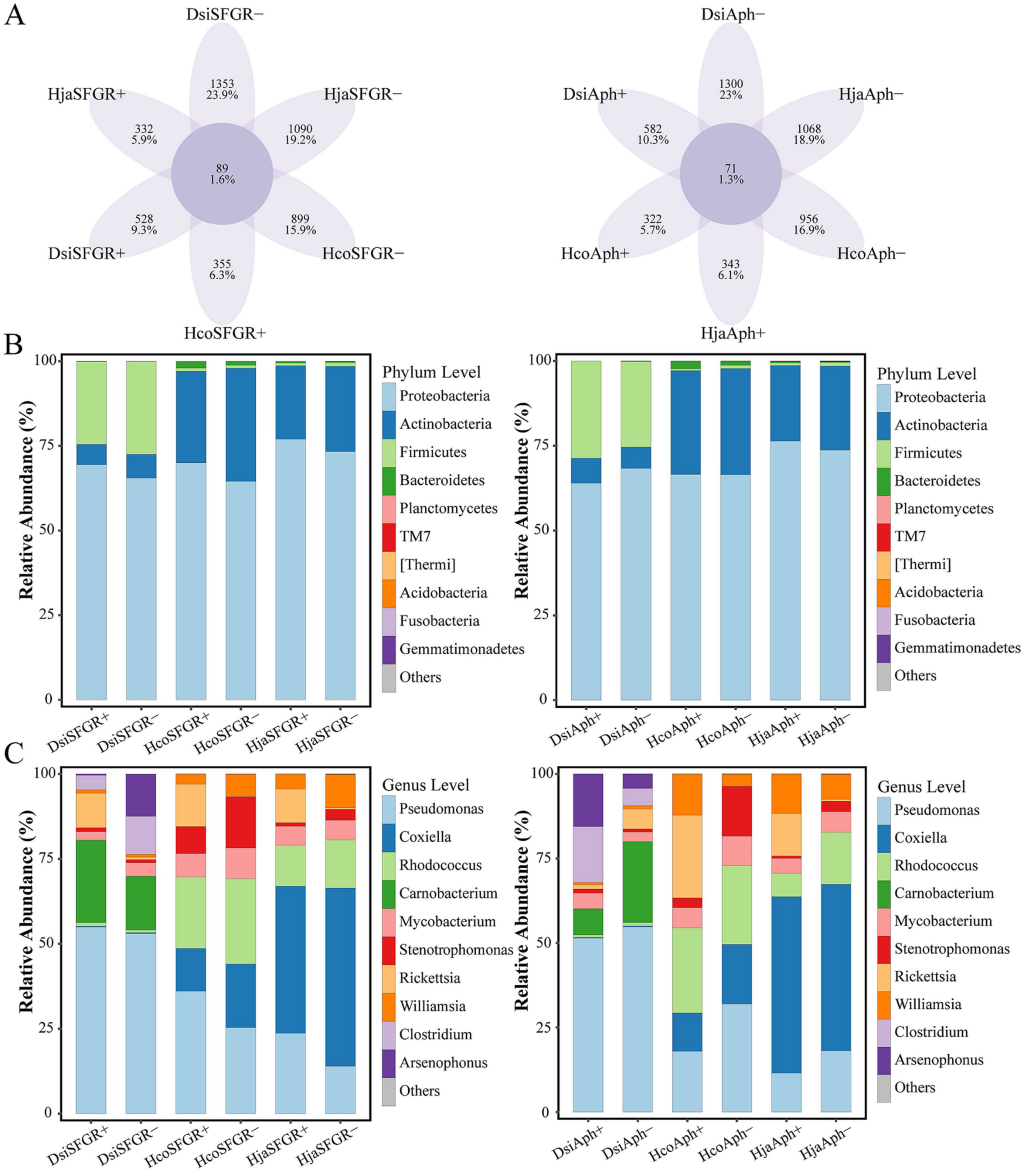
Microbial community divergence across taxonomic ranks in ticks. **(A)** Comparative analysis of unique and shared amplicon sequence variants (ASVs) between pathogen-infected and non-infected groups. **(B)** Phylum-level and **(C)** genus-level differential abundance of microbiota between infected and non-infected groups. The top 10 most abundant phyla **(B)** and genera **(C)** are displayed. Dsi, *Dermacentor silvarum*; Hco, *Haemaphysalis concinna*; Hja, *Haemaphysalis japonica*.

Figure 4A presents an amplicon sequence variant (ASV)-level Venn diagram comparing the microbial composition across infection status groups. The analysis revealed that non-infected groups had more unique ASVs than their pathogen-infected counterparts (mean ± SEM: 1111.0 ± 78.5 vs. 410.3 ± 47.7; Mann–Whitney *U*-test, p < 0.01). This pattern remained consistent across all analyzed tick species, demonstrating enhanced microbial specificity in non-infected arthropods. A total of 5,954 ASVs were classified into 33 phyla, 362 families, and 804 genera. Proteobacteria, Firmicutes, Actinobacteria, and Bacteroidetes were the dominant phyla. In addition, other phyla, including Planctomycetes, TM7, Thermi, Acidobacteria, Fusobacteria, and Gemmatimonadetes, were observed. At the phylum level, Proteobacteria, Firmicutes, and Actinobacteria dominated the microbial profiles of all three tick species (Figure 4B). Notably, *D. silvarum* exhibited a significantly higher abundance of Firmicutes (18.3 ± 2.1%) than the *Haemaphysalis* species. Genus-level profiling identified Pseudomonas, Coxiella, Rhodococcus, and Carnobacterium as core taxa. A notable observation was that *Rickettsia*-infected ticks exhibited a significantly higher relative abundance of Pseudomonas compared to non-infected controls, whereas *Anaplasma*-infected ticks showed an inverse trend with reduced Pseudomonas population (Figure 4C).

### 3.4 Impact of microbiota on vectors

**Figure 5:**
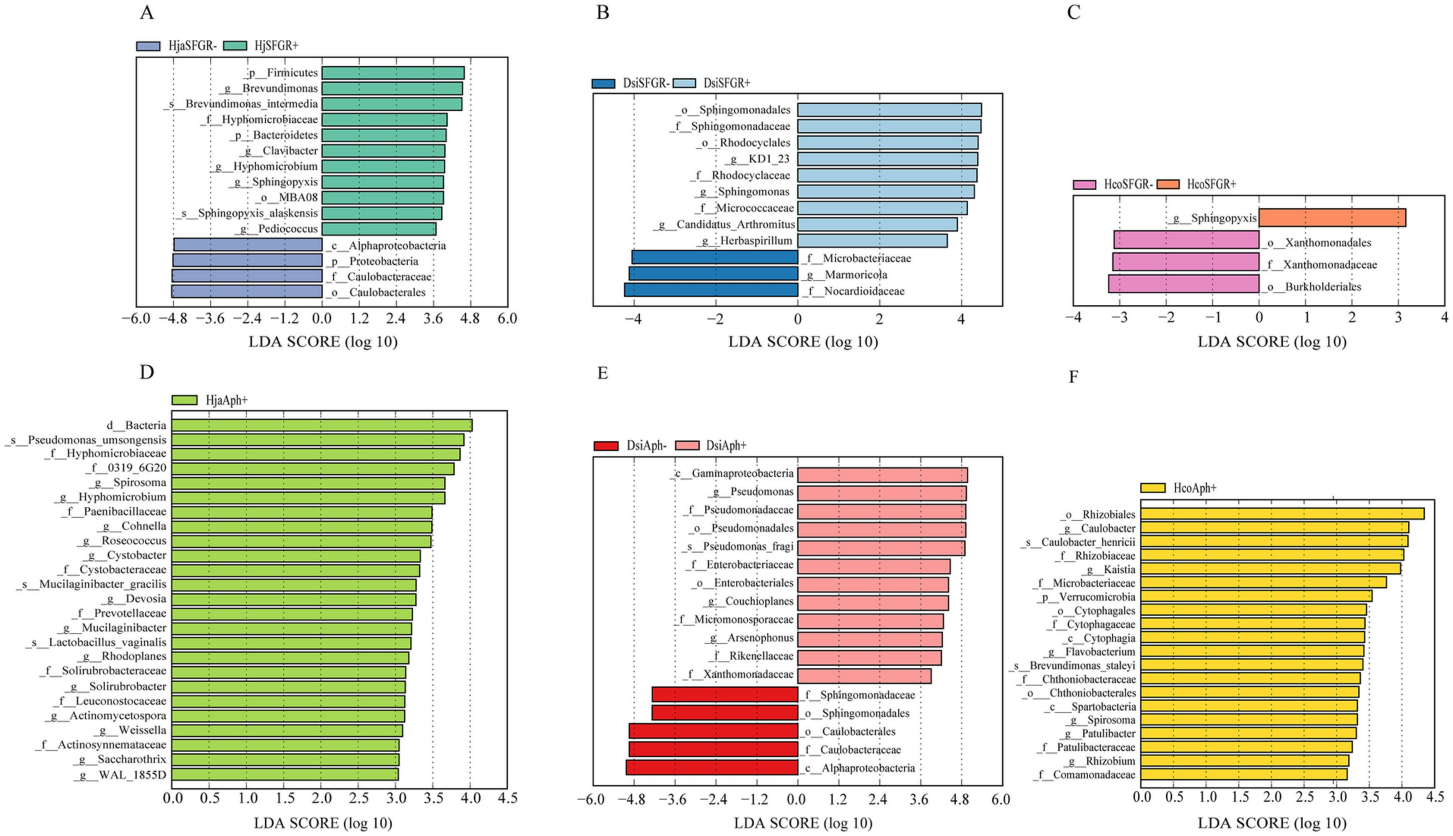
Genus-level biomarkers identified by LEfSE analysis in three tick species infected with distinct pathogens. **(A–F)** Differential genera corresponding to infection of *Haemaphysalis japonica*, *Dermacentor silvarum*, and *Haemaphysalis concinna* with spotted fever group *Rickettsia* or *Anaplasma*. *Dermacentor silvarum*; Hco, *Haemaphysalis concinna*; Hja, *Haemaphysalis japonica*.

LEfSe was employed to analyze how the microbiota varied across groups of different species. The results revealed differences in bacterial community composition between each tick species. In *H. japonica*, LEfSe analysis (LDA—linear discriminant analysis, LDA score >2, *p* < 0.05) identified five significantly divergent taxonomic units at the genus level, while SFGR-infected samples exhibited specific enrichment of microbial taxa, including Brevundimonas, Clavibacter, Hyphomicrobium, Sphingopyxis, and Pediococcus. The results indicate that these taxa are potential biomarkers of infection status. Non-infected samples were predominantly enriched in Caulobacteraceae, implying their antagonistic role in pathogen suppression or host homeostasis (Figure 5A). Notably, *Sphingomonas* and *Sphingopyxis* showed specific enrichment in SFGR-infected *D. silvarum* and *H. concinna*, respectively (Figures 5B–C). When grouped by the *Anaplasma* infection status, the genus of *Spirosoma* emerged as a conserved potential biomarker in both *Haemaphysalis* tick species (*H. japonica* and *H. concinna*). Notably, no significant enrichment of *Spirosoma* was detected in infected *D. silvarum* samples compared to their uninfected counterparts (Figures 5D–F).

## 4 Discussion

In recent years, there has been growing interest in the microbial communities associated with disease vectors. This interest stems from the understanding that bacterial interactions can influence the survival and transmission of pathogens (Wu et al., 2019; Laukaitis and Macaluso, 2021). Despite this, many arthropod-associated human diseases remain undiagnosed; furthermore, our knowledge about the prevalence, diversity, and pathogenicity of novel arthropod-borne pathogens is limited. This highlights the necessity for ongoing microbiological surveillance of vectors. Differences in microbiota composition have been extensively documented in other vector arthropods, including those between vector species, sexes, organs, and different developmental stages (Muturi et al., 2017; Strand, 2018; Choubdar et al., 2021). This study focuses on the differences in microbiota between ticks with regard to two factors: species and pathogens.

Our integrated approach—PCR identification, BLAST alignment, and phylogenetic analysis—revealed two distinct pathogen species: *Anaplasma* and SFG *Rickettsia*. This finding aligns with integrated tick surveillance data from the China-Russia-North Korea border regions, which reported a 35.05% prevalence of *Anaplasma* in five tick species (Min et al., 2025), with phylogenetic clusters showing 99.2–99.7% identity to *A. capra* strains from domestic ruminants in central China (GenBank MT799937, MG869594). Three SFG *Rickettsia* genotypes were concurrently identified, including strains demonstrating 98.4–99.1% sequence homology to Siberian tick isolates (MK304548) and Turkish human clinical variants (MG920563). While these cross-jurisdictional genotypic patterns suggest potential pathogen dispersal across the Tumen River delta, conclusive validation remains constrained by the exclusive sampling of Chinese border territories in existing datasets. *A. capra* exhibits a global distribution pattern (Altay et al., 2022; Numan et al., 2023), with China being both its initial discovery site and major endemic area (Liu et al., 2012). This pathogen infects diverse hosts, including goats, sheep, cattle, and wildlife (Yang et al., 2016; Sahin et al., 2022). Since the first report of human infection in 2015 (Li et al., 2015), its pathogenicity has been confirmed by clinical manifestations such as fever, headache, fatigue, and occasional neurological involvement. *A. capra* strains from China exhibit significant genetic diversity, while retaining >99% 16S rRNA homology with East Asian isolates (Zhang et al., 2020)—a pattern consistent with our phylogenetic analyses, suggesting trans-species transmission networks (Shi et al., 2019; Altay et al., 2024). Intriguingly, South Korean surveillance detected a 17.7% prevalence in *Hydropotes inermis argyropus* (water deer) populations, along with two novel variants (Cheongju and Chungbuk isolates) (Amer et al., 2019). Of particular epidemiological significance, co-infection with *A. capra* and *R. raoultii* was identified in *H. longicornis* parasitizing wildlife hosts, highlighting the complexity of potential mixed pathogen transmission. *R. raoultii* exhibits a pan-Eurasian distribution that is strongly associated with specific tick vectors. Chinese border surveillance confirms its distribution in the analyzed region to be as follows: 32.25% prevalence in *D. silvarum* along the Sino-Russian border (Wen et al., 2014), 6.25% in *H. erinacei* at the Sino-Kazakh border (Guo et al., 2015), and 4% in *D. silvarum* from Mudanjiang, China (Wang et al., 2021). Notably, Northeast China reported two human cases presenting tender eschars without lymphadenopathy (Jia et al., 2014). In South Korea, *H. longicornis* exhibited a remarkably high *R. raoultii* infection rate of 40.9%, with dogs identified as a potential mammalian reservoir. These findings underscore the critical role of this tick species as a zoonotic transmission vector (Seo et al., 2020; Tariq et al., 2021). In Europe (including France, Spain, and Germany), *Dermacentor marginatus* and *Dermacentor reticulatus* serve as the principal vectors, with infection rates ranging from 2 to 80% across studies. Clinical cases typically present with hallmark TIBOLA/DEBONEL manifestations (Parola et al., 2009). The high genetic conservation observed across geographical isolates, as demonstrated by ≥99.4% sequence similarity in the *ompA* gene, underscores their evolutionary stability (Mediannikov et al., 2008). Both *A. capra* and *R. raoultii* demonstrate transboundary endemicity influenced by vector distribution, wildlife reservoirs, and geographic factors, necessitating enhanced multinational surveillance and clinical vigilance.

Our results revealed significant differences in the microbial composition of hematophagous ticks infected by various pathogens. This finding aligns with previous studies that have demonstrated how different pathogens can shape the microbial communities within their vectors, potentially influencing vector competence and disease transmission dynamics (Lacroux et al., 2023; Qiu et al., 2024). The intergroup analysis further elucidated the distinct microbial profiles associated with specific pathogens, suggesting that the presence of certain pathogens may drive the selection of particular microbial taxa. For instance, the dominance of specific bacterial genera in arthropods infected with pathogens such as *Plasmodium* or the dengue virus indicates a potential role for these microbes in modulating the host immune response or enhancing pathogen survival (Wu et al., 2019).

Our integrated analysis revealed that the genus *Spirosoma*—recently reclassified under Mycoplasmatota—showed significant enrichment in *Anaplasma*-infected *Haemaphysalis* ticks (LDA >2, *p* < 0.05), but was undetectable in *Anaplasma*-positive *Dermacentor* cohorts. This host-specific association suggests that the biomarker potential of *Spirosoma* for *Anaplasma* surveillance may be restricted to *Haemaphysalis* ticks. Similarly, *Sphingopyxis* enrichment was only seen in the *Haemaphysalis* species and showed no correlation with *Rickettsia* infection status in the analyzed tick populations (Oren and Garrity, 2021). This bacterium is found in various arthropods and has been extensively documented in ticks (Binetruy et al., 2019a). Spiroplasmataceae is transmitted between arthropods by maternal inheritance and horizontal transfer (Bell-Sakyi et al., 2015).

## 5 Conclusion

This study investigated the prevalence of SFGR and *Anaplasma* in ticks collected from the Yanbian region and examined the changes in the microbiome that occur following infection with these pathogens. The results showed that SFGR and *Anaplasma* had high positivity rates in Yanbian, whereas *D. silvarum* was the tick species with the highest prevalence of infection and the dominant tick species in the region. Notably, certain bacterial taxa were significantly enriched in infected ticks, suggesting their potential role as biomarkers of pathogen presence. The study highlights the complex interactions between tick-borne pathogens and the tick microbiome, providing insights into the ecological dynamics of pathogen transmission. The findings underscore the importance of monitoring tick microbiomes as part of integrated vector management strategies. Future research should focus on elucidating the functional roles of the identified microbial taxa in pathogen transmission and exploring their potential as targets for tick-borne disease control.

## Data Availability

The datasets presented in this study can be found in online repositories. The names of the repository/repositories and accession number(s) can be found at: https://www.ncbi.nlm.nih.gov/bioproject/PRJNA1225783.
