# Supplementary material for: Differential impact of spotted fever group rickettsia and anaplasmosis on tick microbial ecology: evidence from multi-species comparative microbiome analysis
Source: Front Microbiol. 2025 May 13;16:1589263. doi: 10.3389/fmicb.2025.1589263 (PMC12106494; doi:10.3389/fmicb.2025.1589263)
Supplement: Supplementary file 2 [file Table_2.DOCX]

Table S2 PCR primers for ticks and pathogens.

| Pathogen | The name of the gene | Primer sequences (5′-3′) | Fragment size (bp) | Reference |
| --- | --- | --- | --- | --- |
| Spotted Fever  Group *Rickettsia* | ompA | F:ATGGCGAATATTTCTCCAAAA  R:GTTCCGTTAATGGCAGCATCT | 632 | (Roux et al., 1996) |
| *Anaplasma* | 16S rRNA | F:AGAGTTTGATCCTGGCTCAGAACG  R:CACCTCTACACTAGGAATTCCGCTAT | 647 | (Zhou et al., 2010) |
| Tick | 16S rDNA | F:TTACGCTGTTATCCCTAGAG  R:CTGCTCAATGATTTTTTAAATTGCTGTGG | 350 | (Chen et al., 2014) |

Roux, V., Fournier, P.E., and Raoult, D. (1996). Differentiation of spotted fever group rickettsiae by sequencing and analysis of restriction fragment length polymorphism of pcr-amplified DNA of the gene encoding the protein rompa. *J Clin Microbiol* 34**,** 2058-2065. doi: 10.1128/jcm.34.9.2058-2065.1996.<https://doi.org/10.1128/jcm.34.9.2058-2065.1996>

Zhou, Z., Nie, K., Tang, C., Wang, Z., Zhou, R., Hu, S., et al. (2010). Phylogenetic analysis of the genus anaplasma in southwestern china based on 16s rrna sequence. *Res Vet Sci* 89**,** 262-265. doi: 10.1016/j.rvsc.2010.02.009.<https://doi.org/10.1016/j.rvsc.2010.02.009>

Chen, Z., Li, Y., Ren, Q., Luo, J., Liu, Z., Zhou, X., et al. (2014). Dermacentor everestianus hirst, 1926 (acari: Ixodidae): Phylogenetic status inferred from molecular characteristics. *Parasitol Res* 113**,** 3773-3779. doi: 10.1007/s00436-014-4043-1.<https://doi.org/10.1007/s00436-014-4043-1>
